# Social Media in Adolescent Health Literacy Education: A Pilot Study

**DOI:** 10.2196/resprot.3285

**Published:** 2015-03-09

**Authors:** Carrie KW Tse, Susan M Bridges, Divya Parthasarathy Srinivasan, Brenda SS Cheng

**Affiliations:** ^1^The University of Hong KongHong KongChina (Hong Kong); ^2^The University of Hong KongCenter for the Enhancement for Teaching and Learning/Faculty of EducationHong KongChina (Hong Kong)

**Keywords:** social media, health literacy, oral health literacy, dentistry, adolescents, oral health, health informatics

## Abstract

**Background:**

While health literacy has gained notice on a global stage, the initial focus on seeking associations with medical conditions may have overlooked its impact across generations. Adolescent health literacy, specifically in dentistry, is an underexplored area despite the significance of this formative stage on an individual’s approach to healthy lifestyles and behaviors.

**Objective:**

The aim is to conduct a pilot study to evaluate the efficacy of three major social media outlets - Twitter, Facebook, and YouTube - in supporting adolescents’ oral health literacy (OHL) education.

**Methods:**

A random sample of 22 adolescents (aged 14-16 years) from an English-medium international school in Hong Kong provided informed consent. Sociodemographic information, including English language background, social media usage, and dental experience were collected via a questionnaire. A pre- and post-test of OHL (REALD-30) was administered by two trained, calibrated examiners. Following pre-test, participants were randomly assigned to one of three social media outlets: Twitter, Facebook, or YouTube. Participants received alerts posted daily for 5 consecutive days requiring online accessing of modified and original OHL education materials. One-way ANOVA ( analysis of variance) was used to compare the mean difference between the pre- and the post-test results among the three social media.

**Results:**

No associations were found between the social media allocated and participants’ sociodemographics, including English language background, social media usage, and dental experience. Of the three social media, significant differences in literacy assessment scores were evident for participants who received oral health education messages via Facebook (*P*=.02) and YouTube (*P*=.005).

**Conclusions:**

Based on the results of the pilot study, Facebook and YouTube may be more efficient media outlets for OHL promotion and education among adolescent school children when compared to Twitter. Further analyses with a larger study group is warranted.

## Introduction

### Background

The notion of “health literacy” (HL) has established itself in the health care literature in the past 30 years [1] with increasing efforts made at adapting the concept to dental practice and oral health care research [2-10]. “Oral health literacy” (OHL) has, therefore, been defined as “the degree to which individuals have the capacity to obtain, process and understand basic oral and craniofacial health information and services needed to make appropriate health decisions” [11]. Various instruments have been developed to measure the oral health literacy levels of individuals [12] as related to specific conditions such as early childhood caries [13] and periodontal problems [14]. Little work, however, has been undertaken to explore associations between HL across generations, especially adolescent populations.

### Adolescence and Health Literacy

While low health literacy is prevalent among all age groups, adolescents, to a more limited extent than adults [15], have been indicated as a group worthy of particular attention, with little research on this topic conducted, to date, in secondary school environments [16]. The motivation to address adolescent HL can be viewed in chronological terms given that adolescents are both current “dependent users” as well as the future “independent users” of the health care system [16]. As such, adolescence is a crucial period for adapting lifelong health behaviors and habits and may be a key juncture for HL interventions supporting informed, health-seeking lifestyles across adulthood. Existing studies have established that low adolescent literacy/health literacy is associated with risky behaviors including tobacco use and aggression [17], obesity [18], and lower levels of health-promoting behaviors [19]. As health systems increasingly rely on internet usage and, as adolescents are the early adaptors of new technologies, the suggestion that access to online health services, presents another medium to build health literacy [20] could be relevant to this specific group.

### Social Networking Websites

The increasing dependence of patients on social media websites as a source of health care information indicates a need for future studies to assess their impact on health care utilization and outcomes [21]. Emerging social media websites play a vital role in online health searches [22]. Studies in the United States have revealed high usage of Web 2.0 social media sites such as Facebook, YouTube, Twitter, MySpace, and Second Life among Americans aged 18-30 years with two thirds of the population claiming to have visited these sites frequently [23,24]. Facebook has continued to expand its features to compete with instant sharing of videos, images and text updates. For example, during the 2008 US election, almost 8% of people polled under age 30 became an online Facebook “friend” of one of the presidential candidates [25].

With more than 100 million videos being viewed on YouTube everyday [26], researchers are recognizing that YouTube holds value for personal health decision-making [27]. Twitter is another highly prominent and widely used medium with approximately 572,000 new accounts created in the month of March 2011 alone [28]. One recent study has used Twitter as a real-time avenue for monitoring public health, specifically for non-medical use of the psycho-stimulant drug, Adderall, among college students [29]. The effect of social media on oral health is under-researched, however, the available evidence demonstrates the importance of social media as a health education tool. Recent work has called for oral health care professionals to recognize the importance of social websites in shaping public opinion about their profession [30].

### Adolescents and Media Literacy

Researchers have emphasized the need for empowerment education, particularly among children and adolescents [31-33], as an effective health education and prevention model for personal and social change [34-37]. Literature on media literacy and prevention has also demonstrated the importance of critical thinking skills for adolescents to make proper use of media and reduce health risk-taking [37-39]. Bergsma’s 2004 analysis of media literacy and the American Legacy Foundation’s Truth Campaign on tobacco misrepresentations indicated how younger generations “can be powerful advocates for social change through use of the media” [37]. In addition, she recommended that the primary focus of both media literacy and health promotion programs should be to identify the social concerns of youth in order to help them to channel their fresh perspective, unique energy and creativity toward accomplishing social change [37].

### Life Course Analysis Theory for Social Determinants of Health

In addressing the need to examine health literacy and social media, this project has taken into account life course analysis theory into the social determinants of health. This approach focuses on individual levels and seeks to explore cognitive and affective processes determining behavior and lifestyle [38]. Research indicates that the stage of transition from primary to secondary school is a critical period in determining the health status of individuals and levels of health inequalities [39]. Life course analysis, therefore, places its focus on social context and the interaction between people and their environments in the passage through life [38]. Drawing on this theory, this study further explores the “importance of timing” and identifies “windows of opportunity” for adolescents [40] by conducting an oral health literacy intervention utilizing three major social media outlets, Facebook, Twitter and YouTube, with secondary school students. A core research question was, which social media platform has a greater impact on adolescents’ development of oral health literacy? Ultimately, the goal of this study is to produce greater understandings for health promotion providers on how to better utilize social media to disseminate health messages for the purposes of preventative and long-term benefits for adolescents and their future oral health.

## Methods

### Recruitment

This was a cross-sectional pilot study conducted on a random sample of 22 English-speaking adolescents (aged 14-16 years) recruited from grades 9 and 10 in an English-medium international school in Hong Kong. The parents were contacted through the school with an explanation of the objectives of the study and written informed consent was obtained. Participation was voluntary and no additional efforts were made to enroll the participants. Eligibility criteria included healthy, English-speaking participants who were 14-16 years old. Each social medium was randomly assigned to the study population in order to reduce the probability of bias. This study was approved by the Institutional Review Board of the University of Hong Kong/Hospital Authority Hong Kong West Cluster (UW-12-385).

### Materials

Two pre-trained examiners independently conducted the pre- and post-test oral health literacy assessments (REALD-30) and a good inter-examiner agreement was found with Kappa statistics [41]. As a validated word recognition instrument, REALD-30 was developed particularly for the oral health context [7]. It is interview-based, requiring participants to read aloud a list of 30 oral health-realted words arranged in increasing order of difficulty ( time is 2 minutes).

For the OHL education phase, participants received alerts posted daily for 5 consective days requiring online accessing of the modified and original OHL education materials which were reviewed by an expert panel. The online oral health education materials originated from multiple sources: public information provided by the Hong Kong Department of Health [42], educational websites, YouTube videos, and original materials developed and reviewed by an expert panel for this study. These were, therefore, seen as relevant to the target group. Any adaptations of existing online materials were based on the modality of the target social networking medium (ie, text-only-Twitter, video-YouTube, and still images and text-Facebook). Each message posted on the particular social media outlet contained the same principal message.

### Statistical Analysis

Data analyses were carried out using the Predictive Analytic Software (PASW) statistics version 18.0. The inter-examiner reliability was assessed with Kappa statistics. Descriptive statistics were produced to examine the profile of the study group followed by one-way Analysis of Variance (ANOVA), a statistical method used to test the differences between two or more means. Two sets of analyses were undertaken. First, significant differences of the mean between the three social media groups with respect to participants’ sociodemographic characteristics were examined with Fischer’s exact test. Second, the mean difference between the pre- and the post-tests of participants among the three social media groups were compared.

## Results

### User Statistics

Table 1 presents the sociodemographic profile of the study group. After random allocation, there was equal distribution of participants in both Grade 9 and 10 who received YouTube and Twitter, whereas 75% of Grade 9 students received Facebook. The average age of participants was approximately 14-15 years; the ratio of boys to girls was slightly higher in the YouTube group. More than 87.5% of the group’s parents were employed with a monthly family income of over HKD 40,000. Of the participants, 60% were born in Hong Kong with predominantly 90% being of Chinese ethnicity. There was significant difference found with participants’ birth country (*P*=.02). Participant’s average years of speaking English were mixed but over 50% reported having spoken English for 5-10 years. Over 60% of the subjects reported regular past dental attendance.

**Table 1:** Sociodemographic profile of the study group (n=22).

		Twitter (n=6)			Facebook (n=8)		YouTube (n=8)		*P* value^d^
		n		Valid %	n	Valid %	n	Valid %	
**Grade**									
	Grade 9	4		66.7	6	75.0	4	50.0	0.66
	Grade 10	2		33.3	2	25.0	4	50.0	
**Age**									
	14 years old	3		50.0	6	75.0	4	50.0	0.83
	15 years old	2		33.3	2	25.0	3	37.5	
	16 years old	1		16.7	-	-	1	12.5	
**Gender**									
	Male	3		50.0	4	50.0	5	62.5	1.00
	Female	3		50.0	4	50.0	3	37.5	
**Parent’s employment**									
	Unemployed	-		-	1	12.5	-	-	1.00
	Employed	6		100.0	7	87.5	8	100.0	
**Country of Birth**									
	China	3		50.0	-	-	-	-	0.02
	Hong Kong	3		50.0	7	87.5	7	87.5	
	Others	-		-	1	12.5	-	-	
**Ethnicity** ^a^									
	Chinese	1		100.0	7	87.5	1	100.0	1.00
	Others	-		-	1	12.5	-	-	
**Years of speaking English**									
	5-10 years	5	83.3		3	37.5	6	75.0	0.22
	11-16 years	1	16.7		5	62.5	2	25.0	
**Parent’s income** ^b^									
	<30000HKD	-	-		-	-	-	-	1.00
	30000-40000HKD	-	-		1	14.3	-	-	
	>40000HKD	6	100.0		6	85.7	6	100.0	
**Dental experience**									
	Irregular	2	33.3		-	-	2	25.0	0.28
	Regular	4	66.7		8	100.0	6	75.0	
**Social media experience**									
	Seldom	-	-		1	12.5	1	12.5	0.32
	Sometimes	1	16.7		1	15.4	-	-	
	Often	1	16.7		5	62.5	4	50.0	
	Always	4	66.7		1	12.5	3	37.5	

^a^12 participants did not complete this section

^b^3 participants did not complete this section

^c^1USD = 7.76HKD

^d^ *P*-value of 2-sided Fisher’s exact test (in 2 decimal places); Fisher’s exact test was used to compare the proportions among the 3 different media groups.

### Evaluation Outcomes

The self-reported social media experience was mixed between the three social media groups (Table 1). One-way ANOVA between the three groups revealed no significant associations between the social medium allocated and participants’ sociodemographics, including English language background, social media usage, and dental experience. The descriptive statistics of the pre- and post-test scores are shown in Table 2. Of the three social media, higher literacy scores were found in subjects who recieved oral health education messages via Facebook (*P*=.02) and YouTube (*P*=.005). There was high inter-examiner reliability with the kappa value as 0.81.

**Table 2:** REALD-30 scores pre- and post-test (score out of 30).

	Pre-Test				Post-Test				*P* value^a^
	Mean	SD	Min	Max	Mean	SD	Min	Max	
Twitter	16.17	1.83	14.00	19.00	18.00	3.90	15.00	25.00	.32
Facebook	14.50	5.40	7.00	22.00	17.38	4.62	13.00	24.00	.02
YouTube	14.50	5.42	7.00	21.00	18.63	3.42	14.00	24.00	.005

^a^One-way ANOVA

## Discussion

### Principal Results

The results of this pilot study indicate that the social media websites YouTube and Facebook may be more effective in increasing the levels of oral health literacy among adolescents when compared to a short, text message format such as Twitter. This may suggest that the additional audio-visual delivery of health education may improve (oral) health literacy levels more than a solely text-based medium. New Web 2.0 technologies have afforded multimodal literacies and ways to learn with multiliteracy research indicating that students engaged in learning that incorporates multimodal designs, on average, outperform students who learn using print-based, single modes only [43]. Our results support this work as those participants who received messages from Facebook and YouTube with visual plus text or audio-visual information scored significantly higher than text-based Twitter. Indeed, new instruments are embracing this need to assess multi-literacies within health literacy research [44]. However, due to the small number of participants in the pilot study, a larger sample size would make this implication more convincing.

### Life-Course Analysis

In addition to highlighting the utility of social media for health literacy interventions and assessments, the results of this study support the life course analysis theory of the social determinants of health. Life course analysis is a sociological framework emphasizing that life decisions and behaviors are shaped by age, social structures, and historical change [45]. Facebook, as one of the most popular social networking sites is a new historical change in how we interact socially. Life course analysis also places focus on social context and the interaction between people and their environments in the passage through life [38]. The opportunity to increase engagement and interaction of digitally-connected adolescent learners through social media signals potential benefits for improving adolescent literacy [46].

### Comparison With Prior Works

This is a pilot study to explore the effects of social media on the oral health literacy levels of adolescents. The literature indicates that social networks and participatory videos with medical and dental content have started to gain influence in opinion formation by the members of the general public [40,47-51]. The importance of the wide availability and potential influence of YouTube videos regarding dentistry were emphasized in the study by Knosel et al which found that “education videos have a higher degree of usefulness and informational value for laypersons, dental students, and dental professionals than those found in the broader search category” [30]. A recent review has emphasized the importance of social media for disease surveillance concluding that “the growing evidence base regarding the utility of social media for disease surveillance will hopefully encourage academia, industry, the public service, and international organizations to consider social media in a serious light, particularly as a means of engagement rather than just disseminating information” [52].

### Limitations

The study’s results have to be considered in light of its limitations. First, this was a pilot study with a smaller sample size. The sociodemographic variations in the sample were not apparent. More work with larger sample sizes would support further exploration of the influence of sociodemographic variations and the effects of social networks on the oral health literacy levels of adolescents. Second, the participants were recruited from a high socioeconomic status group (monthly income ≥HKD 40,000). This could induce the probability of bias, if one is to infer increased access to technology and these social media because of financial status [53]. Third, this is cross-sectional study, and it would be difficult to establish causality if another time frame was chosen. Finally, the use of a simple word recognition instrument REALD-30 might also be a source of bias, because of its inability to measure the other dimensions (numeracy, reading comprehension, and conceptual knowledge) of oral health literacy [12,44]. However, the study has its own strengths both in terms of innovative material design and target population as well as its attention to the digital aspects of health literacy [53]. The result of future studies with larger sample sizes will also enable health care providers and educators to tailor simplified oral health education materials.

### Conclusions

Based on the preliminary results of this pilot study, it has been possible to conclude that the audio-visual social media of Facebook and YouTube may be more efficient for oral health promotion amongst a sample of adolescent school children when compared to a simple text-based medium such as Twitter.

## Multimedia Appendix 1

Oral health educational materials.

## Abbreviations

ANOVA: analysis of variance
HL: health literacy
OHL: oral health literacy
REALD-30: Rapid Estimate of Adult Literacy in Dentistry
