# Supplementary material for: Social Media in Adolescent Health Literacy Education: A Pilot Study
Source: JMIR Res Protoc. 2015 Mar 9;4(1):e18. doi: 10.2196/resprot.3285 (PMC4376152; doi:10.2196/resprot.3285)
Supplement: Supplementary file 1 [file resprot_v4i1e18_app1.pdf]

e-Appendix: Oral Health Educational Materials

REALD-30 Terms	Twitter	Facebook	YouTube
Sugar	Chewing sugarless gum helps stimulate saliva to fight the bacteria which forms within 5 minutes of eating any sugars! #snackless Smoking #prevent	Chewi 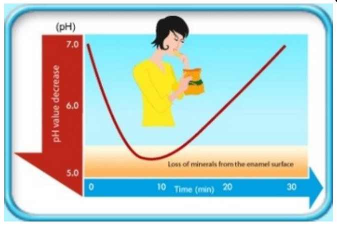 ng sugarless gum helps stimulate saliva to fight bacteria that forms within 5minutes ofeating any sugars! Saliva neutralizes the bacterial products and helps rinse away food particles, too.	https://www.youtube.com/watch?v=qoriw_iR0Kw
Smoking	Not only does smoking affect your general health, smokers are 5x more likely to develop gum disease than nonsmokers! #oralhealth+health	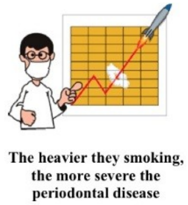 Smokers are 5x more likely to develop gum disease. Your mouth is the window to your overall health so be informed of the risks of smoking before starting.	https://www.youtube.com/watch?v=_r9V4YbnQd0

Floss	Flossing at least once a day in between your teeth helps your gums stay healthy! #oralhygiene #obvious	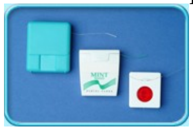 Flossing at least once a day in between your teeth helps remove plaque and keeps your teeth healthy!	https://www.youtube.com/watch?v=KNw65vGnV5U
Brush	Build healthy habits when young. Brush your teeth twice a day! #obvious #oralhealth	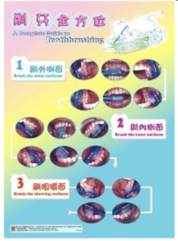 Brush your teeth systematically!	https://www.youtube.com/watch?v=KNw65vGnV5U
Braces	If ur teeth r growing unevenly, a corrective appliance might be recommended, the most common being braces! #orthodontics	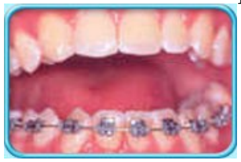 If your teeth are growing in unevenly or in a pattern not best for your mouth a corrective appliance might be recommended, the most common being braces! It is one of the treatments an orthodontist may use to	https://www.youtube.com/watch?v=HCeKQFNyZWk

		guide your teeth back into place.	
Pulp	Did u know that teeth are not dead bone? The most inner core of the tooth is the pulp which has nerves & blood vessels to keep the tooth alive!	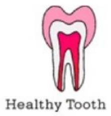 Did you know that teeth are not dead bone? As mentioned previously, the most outer part of the tooth is enamel. The next layer is the dentine. The most inner, sensitive core of the tooth is the pulp, which has nerves and blood vessels to keep the tooth alive.	https://www.youtube.com/watch?v=Hv4pg1P77DI
Denture	Do your grandparents have dentures? Remind them not to wear false teeth overnight so as to avoid fungal infections! #stayaware #family	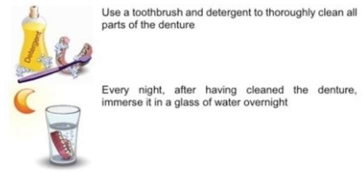 Inform your grandparents of good denture hygiene! Follow these instructions to avoid possible fungal infections.	https://www.youtube.com/watch?v=asx-b4xGRRw

Enamel	Enamel is the outer surface of the tooth. It is hard but can still decay bc of plaque! Saliva helps bring minerals to keep it strong! #brush	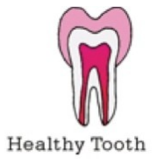 There are different parts to a tooth: enamel, dentine & pulp! Enamel is hard but can still break down if you let plaque accumulate! Saliva is one of the body's defense to bring minerals to the enamel to keep it strong, neutralize acids and wash away food particles.	https://www.youtube.com/watch?v=QGy4uEWLoow
Sealant	Sealants can be applied to the deep grooves (chewing areas) of your molars to prevent plaque build-up and cavities. #askyourdentist	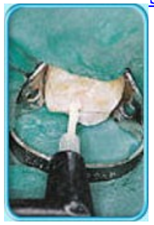 Sealants can be applied to the chewing areas of your teeth to prevent plaque that causes cavities in the deep grooves of your teeth.	https://www.youtube.com/watch?v=2X9Zk5LcJaU
Genetics	Genetics plays a factor in certain dental issues, like your facial profile or future oral and general health problems! #seeyourdentist	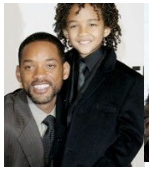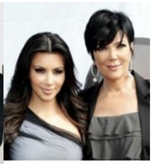 The	https://www.youtube.com/watch?v=iKn8FQa8F34<br>Source: dearholisticdentist

		saying, “Like father like son/like mother like daughter” is true for jaw and tooth development. Genetics plays a factor in certain dental issues, like your facial profile or future oral and general health problems!	
Caries	Tooth decay, also known as dental caries, is most likely to form in the deep grooves of your teeth. #brushaftereating #floss #nosnacks	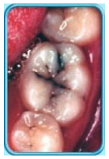 Tooth decay, also known as dental caries, is most likely to form in the deep grooves of your teeth. Avoid formation by brushing after eating, flossing, and avoiding sugary snacks.	https://www.youtube.com/watch?v=_oIlv59bTL4
Restoration	When u have a cavity, u may need a restoration (filling). Ask ur dentist about the different types of filling materials available! #seeyourdentist	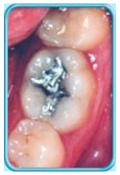 When you have a cavity, you need a restoration (filling). There are different materials that can be used to fill your cavity, like amalgam, which is silver in colour like the picture below.	https://www.youtube.com/watch?v=yWIBrXnoBJk<br>Source: dentistincolumbusga

Fluoride	Application of topical fluoride (available in gel or varnish) on tooth surfaces can prevent caries/reduce tooth sensitivity. #seeyourdentist	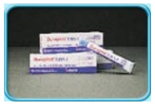 The application of topical fluoride on the tooth surfaces can prevent dental caries or reduce tooth sensitivity. It can be available in gel or varnish.	http://www.youtube.com/watch?v=5w_pz0hi8ew<br>Source: drcivils - Melanie, a dental assistant with Drs. David and Janna Civils talks about different kinds of fluoride
Plaque	Plaque is a biofilm that contains bacteria and if it is not removed could cause periodontal disease and cavities. #oral hygiene #brush	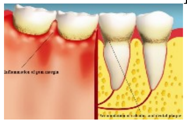 Plaque is a biofilm that contains bacteria and if not removed could cause periodontal disease and cavities.	https://www.youtube.com/watch?v=2X9Zk5LcJaU
Extraction	When your dentist says you need an extraction that means the tooth needs to be pulled out! #prevent #staycalm	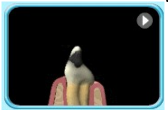 Sometimes if the cavity in your tooth is too extensive, your dentist may say you need an extraction – which means the tooth needs to be pulled out!	https://www.youtube.com/watch?v=NCCH3I12QFI

Periodontal	Bleeding of the gums? You may be suffering from a disease that destroys the gum around the root(s) of a tooth called Periodontal Disease! #seeyourdentist	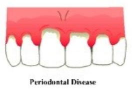 Bleeding of the gums?<br>You may be suffering from a disease that destroys the gum around the root(s) of a tooth called Periodontal Disease!	https://www.youtube.com/watch?v=kWJfL0-d6yY
Fistula	Fistula is an abnormal connection between two areas or vessels that usually do not connect i.e. sinus & oral cavity. #learn	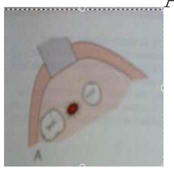 A fistula is an<br>abnormal connection or passageway between two areas or vessels that do not usually connect. For example, an oro-antral fistula can occur as a complication of the extraction of an upper tooth where a pathway is opened between the sinus cavity and the mouth.	http://www.youtube.com/watch?v=cCA_x9ZgU1Q
Cellulitis	Facial infections i.e.cellulitis may become life-threatening if left untreated. It is usually related to problems with wisdom teeth #checkup	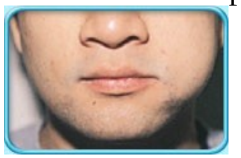 Facial infections<br>such a cellulitis may become life-threatening if the swelling is	http://www.youtube.com/watch?v=ZEVjK3AiEeE

		untreated. Cellulitis infections are more likely to be related to problems with wisdom teeth. Consult your dentist if feeling these symptoms: pain and swelling of the cheek, difficulty in opening the mouth, pain when swallowing, swollen lymph nodes in the neck, fever, and/or bad breath.	
Abscess	A bad tooth infection can cause a pus-filled boil (abscess) in your gum. #seeyourdentist	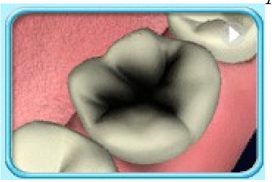 An oral abscess is a true dental emergency.From a bad tooth infection, fluid forms in the jaw bone developing into pus-filled abscess that causes painful swelling. It's like a pimple or boil in your mouth.	https://www.youtube.com/watch?v=YDCbiG9cZBA
Incipient	When the minerals of the outer layer of the tooth are destroyed by acids but has not reached dentine yet, this is known as incipient caries.	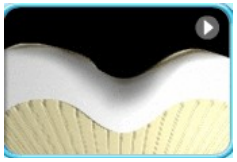 When the minerals of the outer layer of the tooth are destroyed by acids but the bacteria has not caused a cavity and has not reached the inner layer of dentine yet, this is known as incipient	https://www.youtube.com/watch?v=0UfBHb-SOpM

		caries.	
Halitosis	Bad breath is known as halitosis. This can be caused by smoking or if u don’t brush ur tongue, or eat certain foods like garlic&onions!	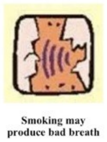 Unpleasant mouth odour is known as halitosis. This can be caused by smoking or if you don’t brush your tongue, or eat certain foods like garlic and onions.	https://www.youtube.com/watch?v=eBcb3bFTAbs
Malocclusion	When your teeth are not in the correct alignment between your upper and lower teeth - that's called malocclusion. #beinformed	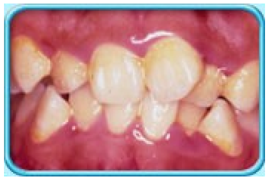 When your teeth are not in the correct alignment between your upper and lower teeth - that's called malocclusion.	https://www.youtube.com/watch?v=ZSxt13wgvHQ
Gingiva	Healthy gingiva (gum) is pink and tightly bound down to a tooth with no unexplained bleeding or swelling. #oralhygiene #brush #floss	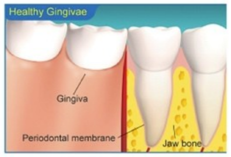 Healthy	https://www.youtube.com/watch?v=KWYDBl29qxo

		gingiva (gum) is pink and tightly bound to your tooth without any unexplainable bleeding or swelling.	
Dentition	A normal set of teeth (dentition) has 32 teeth including 4 wisdom teeth. Types of teeth, includes the incisors, canines, and molars. #learn	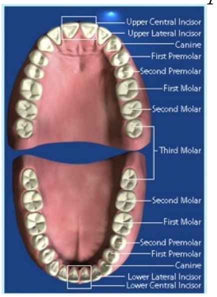 A normal set of human teeth (dentition) has 32 teeth including 4 wisdom teeth. It consists of several different types of teeth, including incisors, canines, and molars.	https://www.youtube.com/watch?v=4ybPifimHqs<br>Source: TutorVista
Bruxism	Teeth grinding (bruxism) causes tooth wear especially when stressed at night. #seeyourdentist #relax	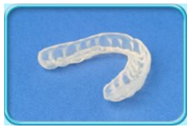 Teeth grinding, also known as bruxism, can be the cause of tooth wear. If severe, you should go to a dentist as soon as possible. The dentist will give appropriate treatment such as fabricating a “mouth guard” to keep the tooth substance from being worn down.	https://www.youtube.com/watch?v=a-igubcZnWs

<table>
<tr><td>Hyperemia</td><td>When the pulp is fighting infection the blood flow increases (hyperemia) to inflamed pulp tissue to deliver immune cells to the area #learn</td><td>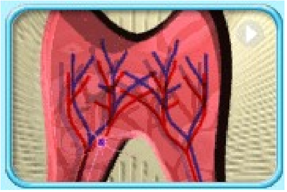 When the pulp is fighting infection the blood flow increases (hyperemia) to pulp tissue (inflamed tissue) to deliver immune cells to the area.</td><td>https://www.youtube.com/watch?v=YDCbiG9cZBA</td></tr>
<tr><td>Analgesia</td><td>You can get over-the-counter products such as Panadol TM for dental pain relief/analgesia. #relief</td><td>You can get over-the-counter products such as Panadol TM for dental pain relief/analgesia. For more severe pain, your dentists can prescribe stronger analgesics.
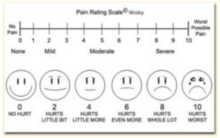
Source: Mosby
Pain Scale</td><td>http://www.youtube.com/watch?v=yyBChwW_RoI</td></tr>
<tr><td>Hypoplasia</td><td>Enamel hypoplasia is defect of the protective enamel which makes the tooth thin and brittle! #cariesrisk #seeyourdentist</td><td>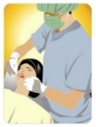 Regular dental check-ups should start from a young age. Your</td><td>http://www.youtube.com/watch?v=vC_kqUs0SAA</td></tr>
</table>

		dentist or hygienist can then identify conditions such as hypoplasia. This thinning of the protective enamel is a likely place for decay to start.	
Apicocetomy	If a root canal fails, you may need an apicoectomy, where they remove the tip to clean the infection at the tip of ur root. #endodontics	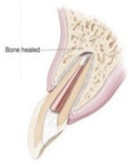 The root of a tooth anchors it into the jaw bone. Infection of the root canal is usually treated by specialist endodontists. If unsuccessful, they may have to remove the tip of the root in a complex procedure called apicocetomy.<br><br>Source: Endodontics in Cranberry – Images by Higher Images - http://www.endointheberry.com/	https://www.youtube.com/watch?v=-f2dxVabmDY
Temporomandibular	Temporomandibular joint is the major joint connecting your lower jaw to the skull. You can feel it move when you chew, talk and yawn #learn	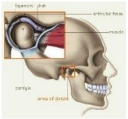 The temporomandibular joint connects your lower jaw (mandible) to the temporal bone at the side of the head. If you put your fingers to the just infront of your ears	https://www.youtube.com/watch?v=KrsRvjNPuGE

and open your mouth you can feel the joint move up and down and side to side which happens when you chew, talk, and yawn.
